# Polyphenol Enriched Diet Administration During Pregnancy and Lactation Prevents Dysbiosis in Ulcerative Colitis Predisposed Littermates

**DOI:** 10.3389/fcimb.2021.622327

**Published:** 2021-06-09

**Authors:** Stefania De Santis, Aurelia Scarano, Marina Liso, Francesco Maria Calabrese, Giulio Verna, Elisabetta Cavalcanti, Annamaria Sila, Antonio Lippolis, Maria De Angelis, Angelo Santino, Marcello Chieppa

**Affiliations:** ^1^ Department of Pharmacy-Drug Science, University of Bari Aldo Moro, Bari, Italy; ^2^ Institute of Sciences of Food Production National Research Council (CNR), Unit of Lecce, Lecce, Italy; ^3^ National Institute of Gastroenterology “S. de Bellis”, Research Hospital, Castellana Grotte, Italy; ^4^ Department of Soil, Plant and Food Sciences, University of Bari, Bari, Italy; ^5^ Department of Pharmacy, University of Salerno, Fisciano, Italy

**Keywords:** ulcerative colitis, nutrition, animal model, polyphenols, microbiota

## Abstract

Neonatal colonization of the gastrointestinal tract depends on mother microbiome, thus mother microbiota dysbiosis is transmitted to the offspring during the delivery and shaped by breastmilk characteristics. Here we used a murine model of UC predisposition (Winnie^-/-^) to evaluate the effects of maternal diet during pregnancy and lactation. Using heterozygous breeders, we obtained both Winnie^-/-^ and C57BL/6 littermates from the same mother and compared their microbiota at weaning and adult age, using a diet enriched with 1% tomato fruit of a line – named Bronze – highly enriched in bioactive polyphenols, or Control tomato. Females received enriched diets two weeks before the beginning of the breeding and never stopped for the following six months. No significant effect was observed in regard to the percentage of Winnie^-/-^ offspring, as with both diets the percentage was about 25% as expected. Winnie littermates from breeders fed with the Bronze-enriched diet showed reduced dysbiosis at 4 weeks of age if compared with Winnie under the Control tomato diet. This effect was then reduced when mice reached adult age. Conversely, the microbiota of C57BL/6 does not change significantly, indicating that fortified mothers-diet significantly contribute to preventing dysbiosis in genetically predisposed offspring, but has mild effects on healthy littermates and adult mice. An overall tendency towards reduced inflammation was underlined by the colon weight and the percentage of Foxp3^+^ cells reduction in Winnie mice fed with Bronze diet. Control diet did not show similar tendency.

## Introduction

Ulcerative colitis (UC) is a relapsing, chronic and debilitating inflammatory disease involving the large intestine from the caecum to the rectum (Ungaro et al., 2017). UC affects both adults and children and peaks during reproductive years. Multiple risk factors are associated with UC development, including environmental factors, eating disorders, emotional distress, immune disorders, microbiota, and genetic predisposition (Corridoni et al., 2014).

Currently, a complete genetic map explaining disease hereditariness is not available, nonetheless, parents’ transmission seems to be the strongest predictor factor for child disease development. Some recent studies suggested that the transmission rate from mothers with Inflammatory Bowel Disease (IBD) is higher than father’s transmission, thus, it is intriguing to speculate a primary role for mothers’ microbiome in IBD transmission (Akolkar et al., 1997; Zelinkova et al., 2012). Intestinal microbial communities play a non-redundant role in shaping the mucosal immune response both directly and indirectly affecting intestinal barrier functions (Takiishi et al., 2017; Torres et al., 2020).

Dysbiosis in IBD patients is characterized by a reduction in microbial diversity compared with healthy patients, increased concentrations of Gram-negative anaerobes and reduced numbers of colonic *Bifidobacterium species* (Wensinck et al., 1981; Matsuda et al., 2000; Ohkusa et al., 2002; Tamboli et al., 2004).

Modulation of the gut microbiota was recently proposed to be an important strategy to protect newborns from chronic disease development (Houghteling and Walker, 2015; Tanaka and Nakayama, 2017; Liso et al., 2018; Turroni et al., 2020). Intestinal bacteria communities adapt to the intestinal lumen content, including dietary antigens. Thus, nutritional strategies are a privileged line of intervention to prevent dysbiosis. More in general, these strategies can dampen inflammation in distinct pathological conditions, especially those involving the use of foods from the Mediterranean diet characterized by a high content in bioactive compounds (De Santis et al., 2019; Piccinin et al., 2019; Cariello et al., 2020; De Santis et al., 2020a). Furthermore, nutrition may shape mothers’ microbiota and breast milk composition, both significantly involved in the development of the offspring intestinal microbial communities (Rautava et al., 2012; Baldassarre et al., 2014; Liu et al., 2014). Thus, nutritional strategies able to promote mothers’ “healthy microbial communities”, may protect the progeny from IBD development.

Examples of maternal diet affecting neonatal health are reported in literature. Breast milk is a source of a vast spectrum of immune-active and antimicrobial molecules, micronutrients, vitamins, antioxidants, and other nutrients, that, altogether, shape the mucosal immune response (Andreas et al., 2015). Furthermore, a direct axis between breast milk composition and maternal intestinal microbiota has been demonstrated, possibly due to the extraintestinal translocation of intestinal bacteria to the mammary gland during late pregnancy and lactation in mice (Perez et al., 2007).

Different studies reported the positive effects of polyphenol-enriched diets on UC in mice, mostly highlighting the preventive potential of their dietary supplementation, even after a short-term administration (2 weeks of diet) (Martin and Bolling, 2015; Wang et al., 2020). The administration of a polyphenols enriched tomato diet has been previously described as an inducer of positive effects on the pathological symptoms and microbiota composition, compared to a standard rodent diet, in IBD mice models (Liso et al., 2018; Scarano et al., 2018). However, the impact of polyphenols-enriched diets on puppies during lactation and later in adult life, in terms of microbiota modulation, was less characterized.

For this reason, we aimed to verify the impact on the microbiota of puppies fed with custom diets supplemented with 1% of lyophilized wild type (Control) or Bronze tomato lines. The near-isogenic Bronze line and its composition have been previously described in detail, and it is based on different classes of polyphenols, such as flavonols, anthocyanins and stilbenoids (Scarano et al., 2018).

The present study showed that maternal diet enriched with lyophilized fruits of a polyphenol-rich tomato line (Bronze) was protective against newborn dysbiosis development. Our previous data demonstrated that 2 weeks of mice chow enriched with 1% of Bronze lyophilized fruits were able to block the inflammatory pathway in the intestinal tract of DSS (Dextran Sulfate Sodium) treated mice (Scarano et al., 2018) and in the genetically predisposed Winnie mice (Eri et al., 2011; Liso et al., 2019). This mutant mouse strain was characterized by a point mutation in the *Muc2* gene, resulting in accumulation of aberrant MUC2 within the intestinal goblet cells, leading to activation of endoplasmic reticulum (ER) stress. ER stress caused the activation of the innate and adaptive immune response, resulting in a spontaneous and severe mucosal inflammation of the distal colon, resembling UC (Eri et al., 2011). Heterozygote mice do not show UC like phenotype, but present mild dysbiosis transmittable to the offspring (De Santis et al., 2021). The breeding strategy was based on co-housing heterozygote breeders to obtain both Winnie^-/-^ and C57BL/6 littermates from the same mother. Maternal transmission has been indicated as the pivotal event for the onset of colonization of intestinal communities; for this reason, we investigated if the differences observed between the fecal microbiome of C57BL/6 and Winnie littermates could still be observed in offspring from the same parents (Liso et al., 2019).

We previously demonstrated that gut microbiota from Winnie mice, fed with standard rodent diet, was significantly different from their wild type siblings, even if they were generated and weaned from the same mother (Liso et al., 2019).

Data from others (Codoñer-Franch et al., 2013: Wall et al., 2013) demonstrated that supplementation with flavonoids during lactation increases the antioxidant properties of breastmilk and reduces inflammatory cytokine production in fetal membranes. Here we demonstrate that a significant path of dysbiosis was present in the fecal material of 4-week old Winnie born from heterozygote mothers compared to wild type C57BL/6 siblings. Vice versa, a similar bacterial composition was detected in the fecal material of 4-week old C57BL/6 and Winnie born from heterozygote mothers when fed with a Bronze-tomatoes enriched diet.

## Material and Methods

### Ethical Considerations

Our investigations were performed under the relevant animal protocol which was approved by Institutional Animal Care Committee of National Institute of Gastroenterology “S. de Bellis” (*Organism* engaged for compliance of *Animal Wellbeing*: *OPBA*). All of the animal experiments were carried out according to the national guidelines of Italian Directive n. 26/2014 and approved by the Italian Animal Ethics Committee of Ministry of Health - General Directorate of Animal Health and Veterinary Drugs (DGSAF- Prot. 768/2015-PR 27/07/2015). All animals were maintained in a controlled environment (20–22°C, 12 h light and 12 h dark cycles, and 45–55% relative humidity).

### Generation of Tomato Lines and Diets

The Bronze tomato line (E8::MYB12, E8::Del/Ros, 35S::StSy) was developed as previously described (Scarano et al., 2018), by sequential crossing of two metabolically engineered lines, Indigo and ResTom. The resulting line, named Bronze because of the metallic brown color of the ripe fruit skin, expressed the genes *AmDelila* and *AmRosea1* that induce anthocyanin biosynthesis, *AtMYB12* regulating flavonol biosynthesis and the gene *VvStSy* for the production of resveratrol and stilbenoids (Scarano et al., 2018).

The Control diet (cv. Moneymaker, wild type) has a same basal composition as a standard rodent diet (Nutrient composition_Amount: Proteins_19.4%; Fats_2.58%; Fibers_5.54%; Ashes_6.76%). The addition of 1% of lyophilized wild-type or Bronze tomato powder was chosen consistently to our previous studies (Liso et al., 2018; Scarano et al., 2018). The addition consists of roughly 0.014 mg of polyphenols/day/mouse incorporated in the Control tomato diet, compared to 0.800 mg polyphenols/day/mouse in the Bronze tomato diet (Scarano et al., 2018).

### Murine Models

C56BL/6 mice were purchased from Jackson Laboratories: (C57BL/6, Stock No.: 000664) while Winnie mice were obtained from the University of Tasmania (Eri et al., 2011). Winnie mice were generated by ENU mutagenesis. A missense mutation in the *Muc2* gene (the base change G -> A) caused the substitution of the Cystein in position 52 into a Tyrosine (G9492A, GenBank accession no. AJ511872).

Four couples of heterozygous breeders, subdivided into two groups, were used. Each group of breeders received a different diet. Freeze-dried tomato was supplemented by addition to a standard rodent diet (4RF18) at 1% (tomato-based-diets: wild type (Control) and Bronze). 6-week old females received enriched diets two weeks before the beginning of the breeding and never stopped for the following six months.

New-born pups were weaned at 4 weeks of age, ear-tagged, and then caged based on sex and similar genotype. For fecal material collection mice were single caged for two hours. Genotype was performed from DNA obtained from 5-mm tail tissues. After the weaning, mice were fed with the same tomato supplemented diets used during the breeding until 16 weeks of age. Body weight, stool consistency, and rectal bleeding were assessed every 4 weeks.

Mice were sacrificed at 16 weeks and colon and mesenteric lymph node (MLN) tissues were explanted to evaluate the clinical severity of colitis. Colon length and weight were measured as indicators of colonic inflammation. The colon/body weight indices were calculated as the ratio of the colon wet weight and the total body weight (BW), and as the ratio of the colon length and the total BW of each mouse.

### Cytofluorimetric Assay

FoxP3 staining: Mesenteric lymph nodes (MLNs) were isolated from mice fed with tomato (Control or Bronze)-enriched food. MLNs were passed through a 30 μm cell strainer (Miltenyi Biotec, Bergisch Gladbach, Germany) to obtain a single-cell suspension and washed with DPBS (Gibco, Waltham, MA, USA) + 0.5% bovine serum albumin (BSA, Sigma-Aldrich, St. Louis, MO, USA).

Single-cell suspensions were stained with CD4-FITC and CD25-PE (Miltenyi Biotec, Bergisch Gladbach, Germany). Cells were then permeabilized with Foxp3 Fixation/Permeabilization Kit (eBioscience, San Diego, CA, USA) and washed with PERM Buffer (eBioscience, San Diego, CA, USA). Finally, cells were stained with Foxp3-APC (Miltenyi Biotec, Bergisch Gladbach, Germany), according to the manufacturer’s instructions.

T cell Intracellular Staining: T cells from MLNs of mice fed with tomato (Control or Bronze)-enriched food were cultured with a 500X Cell Stimulation Cocktail (eBiosceince, San Diego, CA, USA) for 12 h, washed with DPBS + 0.5% BSA and stained with CD4-APC Vio770 (Miltenyi Biotec, Bergisch Gladbach, Germany). After washing, cells were then permeabilized with BD CytoFix/CytoPerm^®^Fixation/Permeabilization Kit^®^ (BD Biosciences, Franklin Lakes, NJ, USA), washed with PERM Buffer, and stained with TNFα-PE and IFNγ-APC according to manufacturer’s instructions (Miltenyi Biotec, Bergisch Gladbach, Germany).

For both stainings, Flow Cytometer acquisition was performed using NAVIOS (Beckman Coulter). At least three experiments were performed. Flow cytometer analysis was performed using Kaluza Software 1.5 (Beckman Coulter, Brea, CA, USA).

### RNA Extraction and qPCR Analysis

Total RNA was isolated from the distal colon of mice fed with tomato (Control or Bronze) enriched food. The RNA was extracted using TRIzol^®^ (Thermo Fisher Scientific, MA, USA) according to the manufacturer’s instructions. Total RNA (1 μg) was reverse-transcribed using an iScript cDNA Synthesis kit (Biorad, CA, USA) with random primers for cDNA synthesis. Gene expression of Gapdh, Tnf, Ifnγ, Il10, Il17a, Il12b, Il6, Hmox1, Slpi, Slc40a1 was assessed using PrimePCR™ SYBR^®^ Green Assays (Biorad, CA, USA; assay ID: qMmuCED0027497, qMmuCED0004141, qMmuCID0006268, qMmuCID0015452, qMmuCID0026592, qMmuCID0022424, qMmuCID0005613, qMmuCID0040051, qMmuCED0004965, qMmuCID0011775, respectively). Real-time analysis was performed on CFX96 Touch System (Biorad, CA, USA) and for the relative expression, the ΔΔCt method was used. At least three experiments were performed.

### DNA Extraction From Stool

Mice were single caged for two hours in a cage without bedding and fecal samples were immediately collected and frozen. Total genomic bacterial DNA was isolated from frozen stool samples of mice using the QIAamp^®^Fast DNA Stool Mini Kit (QIAGEN, Hilden, Germany), according to the manufacturer’s instructions.

### Bacterial Microbiome Estimated by 16S rRNAs Metagenetics and Statistical Analysis

16S metagenetic analyses were carried out at Genomix4life (spin-off of the University of Salerno, Fisciano, Italy) by using the Illumina MiSeq platform. The V3-V4 region of the 16S rRNA gene was amplified for analysis of diversity inside the domains of Bacteria (Klindworth et al., 2013). PCR and sequencing analyses were carried out according to the protocol of Genomix4life.

Bioinformatics analyses of sequence data, from processing of raw DNA sequence reads to alpha index estimates were conducted in QIIME2 (https://doi.org/10.1038/s41587-019-0209-9) microbiome platform (version 2020.8). Paired demultiplexed 16S sequences have been denoised by using q2-deblur QIIME plugin (https://github.com/qiime2/q2-deblur). Taxonomy has been inferred by using the SILVA QIIME-compatible classifier (release 138). Alpha diversity metrics including Shannon entropy and Faith’s PD were also computed by using QIIME2 platform (Shannon, 1997; Chao and Bunge, 2002). Starting from QIIME2 relative abundances, the q2-emperor plugin was used to compute beta diversity metrics.

Significant taxa among groups were computed by using a two side Welch test corrected by multiple test with Benjamini-Hochberg (**Supplementary Metadata**).

After converting the taxa relative abundances (at the genus level) into a presence/absence matrix, Venn diagram were calculated and graphically rendered in R environment by using the ‘VennDiagram’ package (https://cran.r-project.org/web/packages/VennDiagram/VennDiagram.pdf).

For colon/body weight indices, gene expression and FACS analyses, a one-way ANOVA test corrected by using Bonferroni within the Graphpad Prism statistical software (release 5.0), was computed. All data were expressed as means ± S.E.M. obtained from at least three independent experiments.

## Results

### Study Design: Bronze Enriched Diet Administration to UC Predisposed Couples

With the intent to verify the impact of polyphenol enriched diet administration to the offspring of UC predisposed individuals, we created diets supplemented with 1% of lyophilized wild type (Control) or Bronze tomato lines (Scarano et al., 2018).

**Figure 1:**
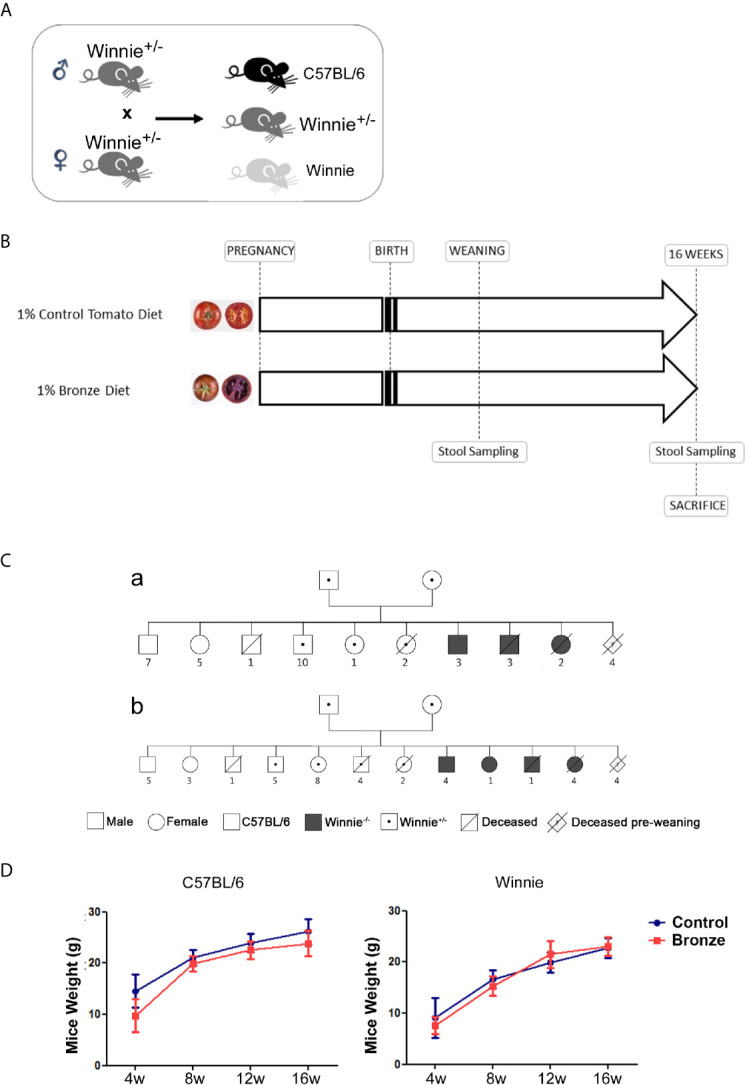
Experimental design. **(A)** Heterozygote Winnie^+/-^ mice were crossed to obtain C57BL/6 and Winnie^-/-^. **(B)** Parental breeders and puppies with the selected genotypes were fed with 1% of Control- or Bronze-enriched diet for up to 16 weeks. **(C)** Family trees of breeding pairs fed with **(a)** Control- or **(b)** Bronze-enriched diet, carried out using the “Pedigree Chart Designer” CeGaT tool. **(D)** Mice weight (male and female) was recorded from weaning at 4 weeks (4 w) to the end of the dietary treatment at 16 weeks (16 w).

**Figure 1A** showed the breeding strategy adopted: C57BL/6 and Winnie puppies were born from the same Winnie heterozygous parents. To study the impact on lactation, Winnie heterozygous breeders were fed with 1% dried fruits of Control tomato variety (cv. Moneymaker) or Bronze tomatoes; the offsprings were fed by lactation until the weaning; afterward, they were fed with the same tomato enriched diets until 16 weeks, when they were sacrificed for further analyses (**Figure 1B**).

Mice were monitored for survival and weight (**Figure 1C** and **Table 1**). **Figure 1C** shows the Family Trees of both experimental groups; siblings have been subdivided by genotype and sex; premature dead mice are also indicated.

**Table 1:** Natality and mortality rates for each experimental group.

DIET	N. LITTERS	N. MICE BORN	N. MICE WEANED	F1 GENOTYPE	N. MICE DEAD
CONTROL TOMATO	7	38	34 (89.5%)	C57BL/6	1
				Winnie^+/-^	2
				Winnie	5
BRONZE	6	42	38 (90.5%)	C57BL/6	1
				Winnie^+/-^	6
				Winnie	5

Both Control and Bronze tomato enriched diets did not affect mice weight, independently from the genotype. However, in line with our models (De Santis et al., 2017; Liso et al., 2019; De Santis et al., 2020b), Winnie mice showed a reduced body weight as compared to C57BL/6 littermates (**Figure 1D**). As expected, the majority of premature deaths were from mice carrying the Winnie allele (in homo- or heterozygosity), regardless of maternal diet. About 90% of the newborn from each experimental group reached adult age.

The mortality rate in the first weeks of life was near 10% in both groups, significantly lower than what we observed in our animal facility with the same breeding strategy, about 20% with Standard rodent diet (data not shown), and the common mortality rate observed in laboratory mice (Weber et al., 2013; Leidinger et al., 2019). As shown in **Table 2**, the obtained percentage for each genotype of the newborns was similar to the predicted one in the Bronze group, whilst in the Control tomato group we recorded an imbalance between the obtained percentage of C57BL/6 and heterozygote Winnie^+/-^ mice.

**Table 2:** Percentages of predicted and obtained genotypes from each breeders couple; total number of siblings for each genotype is shown.

DIET	F1 GENOTYPE	F1% PREDICTED	F1% OBTAINED	N. MICE
CONTROL TOMATO	C57BL/6	25%	38%	13
	Winnie^+/-^	50%	38%	13
	Winnie	25%	24%	8
BRONZE	C57BL/6	25%	24%	9
	Winnie^+/-^	50%	50%	19
	Winnie	25%	26%	10

### Influence of Maternal Diet on Intestinal Microbial Communities of the Offspring

Stools from each mouse were collected at 4 and 16 weeks to carry out a metagenomic analysis of the gut microbiota (**Figure 1B**). We searched for a correspondence in the composition of their microbiota with that of the respective mothers at the weaning.

Both Shannon and phylogenetic Faith’s PD indices were concordant in detecting a higher alpha diversity in all mice (mothers, C57BL/6 and Winnie offspring, at 4 and 16 weeks) fed with Bronze diet when compared with Control tomato fed mice (**Supplementary Metadata** and **Supplementary Table 1**).

In estimating beta diversity, unweighted UniFrac metric reveals how Control tomato and Bronze fed mice do not clearly separate into clusters; two different mixed clouds are visible in a tridimensional plot (**Supplementary Figure 1**).

**Figure 2:**
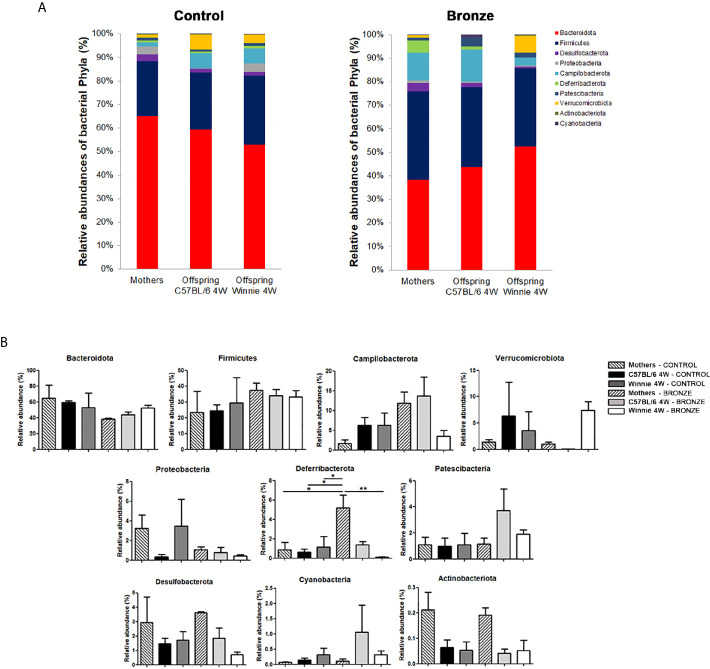
**(A, B)** Relative abundances of the main phyla found at weaning time in the faecal microbiota of mothers and the related offspring (4 weeks), divided in C57BL/6 and Winnie genotypes, fed with Control tomato and Bronze diets. **(B)** *p < 0.05, **p < 0.01.

**Figure 2** reports the comparison of the main phyla found in the mothers and offspring, C57BL/6 or Winnie at 4 weeks. The Bronze diet induced a general trend to an increase of Firmicutes and to a decrease of Bacteroidota, in mothers and related offspring (**Figure 2A**). Campilobacterota tends to increase in mothers and C57BL/6 offspring fed with Bronze diet, while a decrease was observed in Winnie mice (**Figure 2A**). The Bronze diet also induced a tendency of decrease of Proteobacteria in mothers and related Winnie puppies. As shown in **Figure 2B**, mothers fed with the Bronze diet showed a significant increase of Deferribacterota compared to the Control group and the related Winnie offspring.

**Figure 3:**
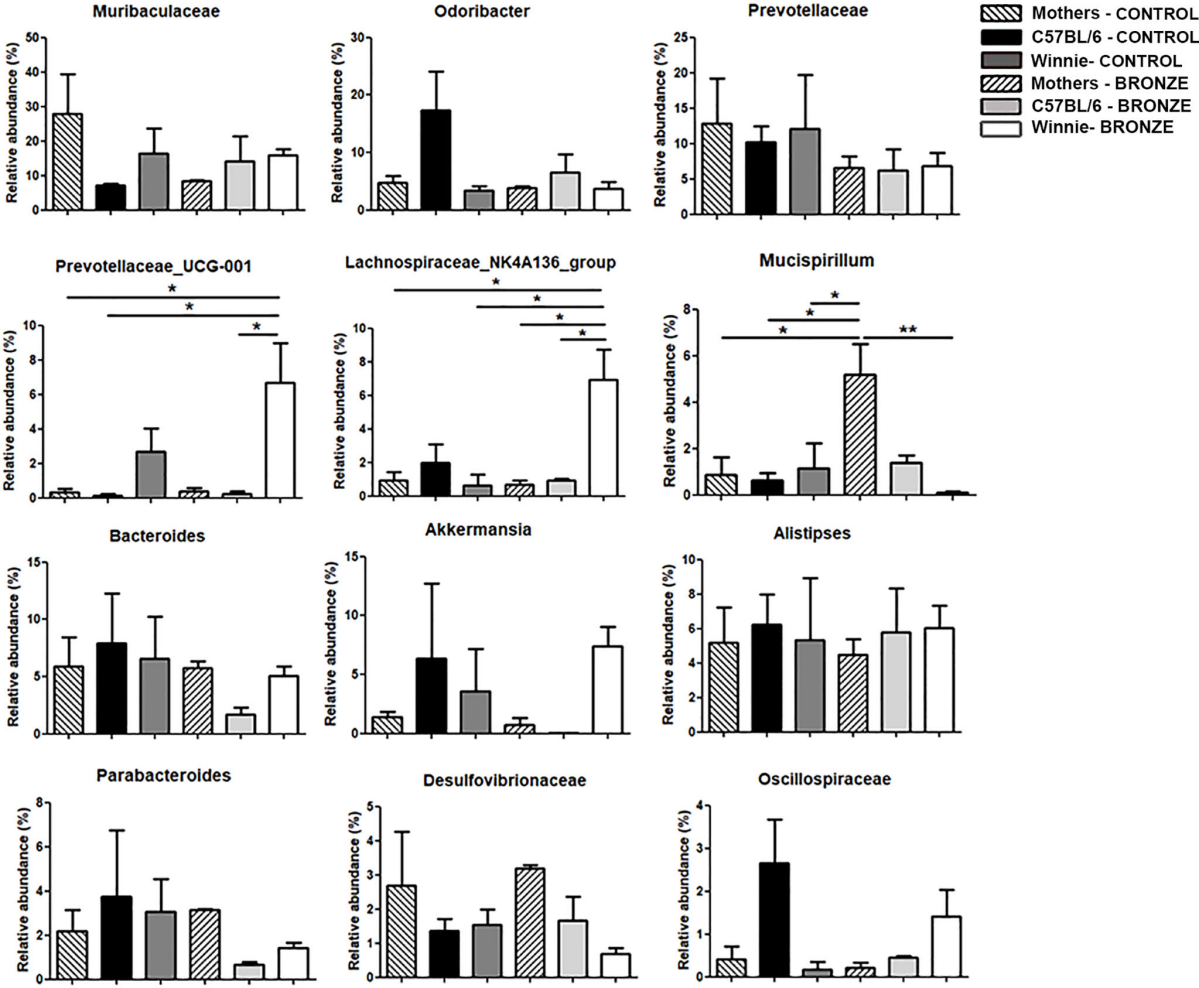
Relative abundances of the bacteria genera found at weaning time in the faecal microbiota of mothers and the related offspring (4 weeks), divided in C57BL/6 and Winnie genotypes, fed with Control tomato and Bronze diets. *p < 0.05, **p < 0.01.

At the genus level, the Bronze diet induced in Winnie mice a significant increase of *Prevotellaceae_UCG-001* and *Lachnospiraceae_NK4A136_group* and a decrease of *Mucispirillum*, compared to Winnie mice fed with the Control diet (**Figure 3**). Instead, in C57BL/6 mice fed with the Bronze diet we observed a decrease of *Odoribacter*, *Prevotellaceae*, *Bacteroides*, *Akkermansia*, *Parabacteroides* and *Oscillospiraceae*, but these differences were not statistically significant (**Figure 3** and **Supplementary Figures 2, 3**).

At the species level, we found a significant increase in *Prevotellaceae_UCG-001;s_spp.* and *Rikenellaceae_RC9_gut_group;s_spp*. in Winnie mice fed with the Bronze diet compared to the Control group (**Supplementary Figure 4**). Such modulations were not detected in C57BL/6 mice after the Bronze diet administration, in which we found a significant decrease of *Odoribacter;s_unidentified* and *Oscillospiraceae;g_uncultured;s_spp*. and a significant increase of *Bacteroides sartorii* and *Mucispirillum schaedleri* (**Supplementary Figure 4**).

### Bronze Diet Administration Is Not Sufficient to Prevent Winnie Associated Intestinal Pathology

**Figure 4:**
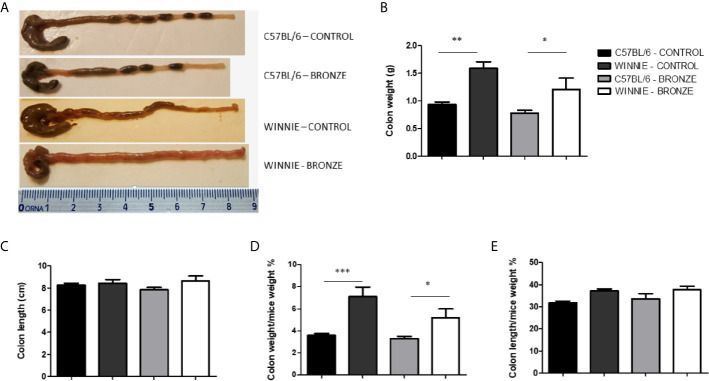
Macroscopic features and measurements of colonic parameters at the end of treatment (16 weeks). **(A)** Representative images of whole colons for each experimental group. Panel **(B, C)** indicate colon weight and length, respectively. Colon weight/body weight and colon length/body weight indices (%) are shown in panel **(D, E)**, respectively. *p < 0.05; **p < 0.01; ***p < 0.001.

After the weaning, C57BL/6 and Winnie mice were fed with the same diets used during the breeding (Control or Bronze enriched diet) until 16 weeks of age. **Figure 4** showed that, at a macroscopic level, no differences were observed in C57BL/6 mice fed with both diets in terms of colon length and weight. On the contrary, the Bronze diet tend to reduce the absolute and relative colon weight in Winnie mice (**Figures 4B, D**), even if the typical phenotype of Winnie was maintained, as they showed watery stools and a higher colon weight than their C57BL/6 littermates (**Figures 4A, B, D**).

**Figure 5:**
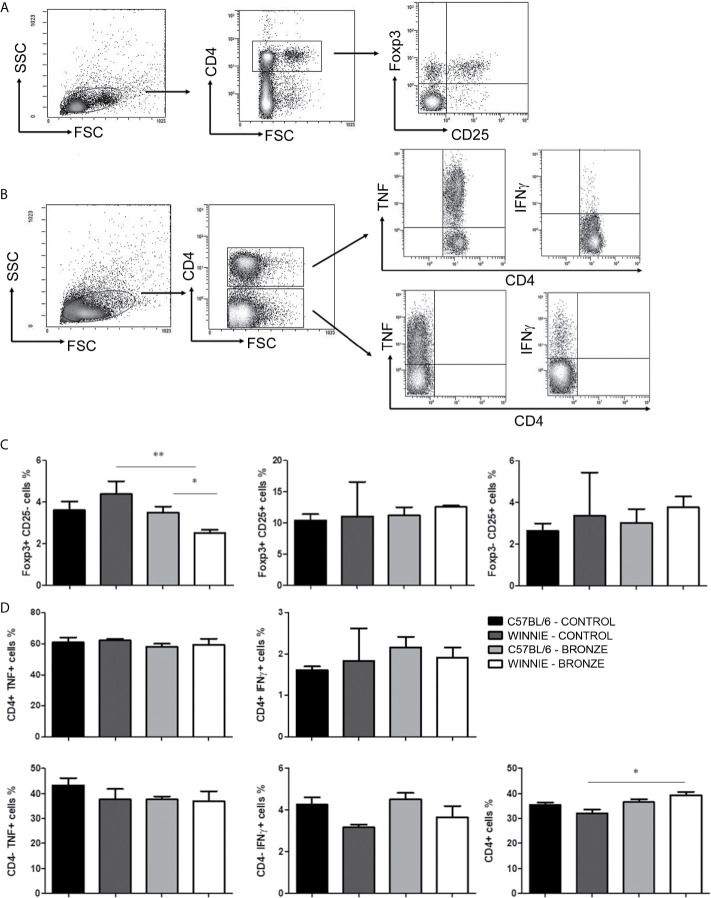
Treg and intracellular cytokine staining of CD4^+^ and CD4^-^ cells isolated from MLNs. **(A)** Representative density plot of CD4-CD25-Foxp3 staining on Treg. **(B)** Representative density plot analysis of intracellular staining of TNF and IFNγ from T CD4^+^ and CD4^-^ cells. **(C)** Histograms represent the percentages of Foxp3 and CD25 gated on CD4^+^ cells, in the MLNs of C57BL/6 mice fed with Control or Bronze diet (black and dark grey bars) and Winnie (light grey and white bars, respectively). **(D)** Intracellular staining of TNF and IFNγ gated on CD4^+^ and CD4^-^ cells, and percentage of total CD4^+^ cells isolated from MLNs of C57BL/6 and Winnie mice, fed with Control or Bronze diet. *p < 0.05; **p < 0,01.

We then isolated T cells from mesenteric lymph nodes (MLNs) and compare the Treg population and the intracellular cytokines produced by T helper (Th) cells in all the experimental groups, following the protocol described in **Figures 5A, B**, respectively. **Figure 5C** showed that the Bronze diet significantly reduced the percentage of CD4^+^ Foxp3^+^ cells in Winnie mice relative to C57BL/6 Bronze-fed and Winnie Control-fed mice. Instead, no significant differences were observed in the intracellular staining performed on Th cells, but we detected an increasing tendency in the CD4^+^IFNγ^+^ cells in C57BL/6 fed with the Bronze diet, as previously observed in adult C57BL/6 mice fed with the Bronze diet for 2 weeks (**Figure 5D**). The Bronze diet also induced a significant increase in total CD4^+^ cells in Winnie mice (**Figure 5D**).

To investigate the influence of the two diets at the molecular level, we analyzed the relative expression of genes involved in the inflammatory response in the colon of C57BL/6 and Winnie mice (**Supplementary Figure 5**). The Bronze diet induced a significant increase of *Ifnγ* expression in C57BL/6 mice, confirming the intracellular staining data on Th cells isolated from MLNs; on the contrary, a tendency for a reduction was observed for *Il6* (statistically significant), *Il10*, *Il12b* and *Il17a* expression. In Winnie mice fed with the Bronze diet, instead, we recorded a tendency for a non-significant increase of *Il6*, *Il10* and *Il17a* expression and a tendency for a decrease of *Il12b*. No relevant difference was observed in *Tnf* expression level in C57BL/6 and Winnie mice fed with both diets. We previously demonstrated that polyphenols like quercetin are able to induce the expression of genes involved in iron metabolism. In Winnie mice, the Bronze diet tends to increase for the expression levels of *Slpi* and *Hmox1*, while reduces the expression of *Slc40a1* (encoding Ferroportin). A similar trend was also observed in C57BL/6 mice but with a significant reduction of *Slc40a1* expression (**Supplementary Figure 5**).

### Long Term Administration of Bronze Diet Is Not Able to Treat Winnie Associated Dysbiosis

**Figure 6:**
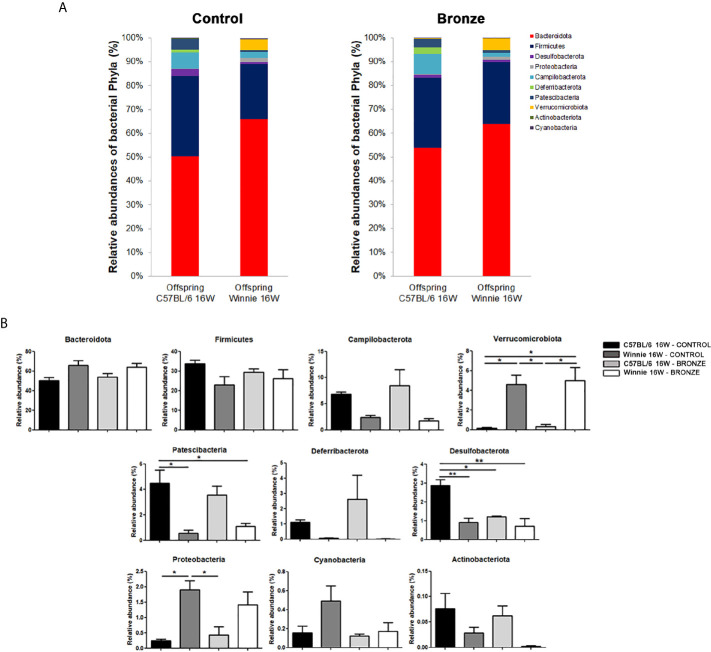
**(A, B)** Relative abundances of the main phyla found in the faecal microbiota of the C57BL/6 and Winnie mice fed with Control tomato and Bronze diets for 16 weeks. **(B)** *p < 0.05; **p < 0.01.

**Figure 7:**
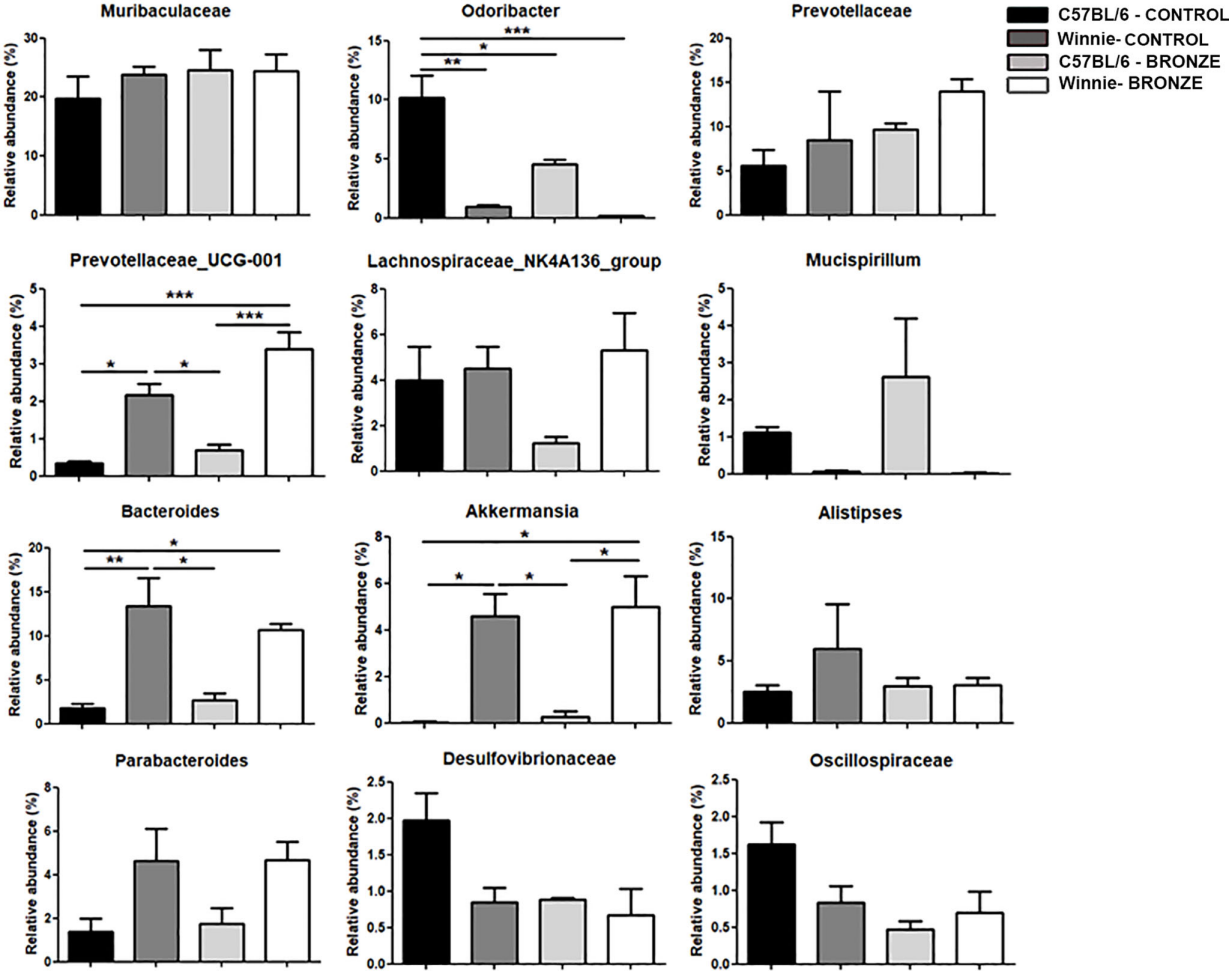
Relative abundances of the main genera found in the faecal microbiota of the C57BL/6 and Winnie mice fed with Control tomato and Bronze diets for 16 weeks. *p < 0.05; **p < 0.01; ***p < 0.001.

We then studied the long-term effects of the polyphenol enriched diets, by comparing the microbiota of 16-weeks old mice at the end of treatment. **Figure 6** showed that the main differences observed at the weaning were more attenuated in older mice. In fact, within the same genotype, the relative abundance of the main phyla was similar with both diets. In the Control tomato groups, the relative abundances of the major phyla, such as Firmicutes, Bacteroidota and Campilobacterota, were almost comparable to those of the offspring fed with Bronze diet. On the other hand, these latest could be comparable to the relative abundances already observed at 4 weeks. In C57BL/6 mice, the Bronze diet induced a decrease of Patescibacteria and Desulfobacterota (significant) and a concomitant increase in Verrucomicrobiota, Deferribacterota and Proteobacteria (**Figure 6B**). In Winnie mice, instead, the Bronze diet induced an increase of Patescibacteria and a decrease of Proteobacteria, Cyanobacteria and Actinobacteriota (not statistically significant, **Figure 6B**). The attenuation of the differences regarding the phyla relative abundances was mostly reflected in the changes at genus (**Figure 7** and **Supplementary Figure 6**) and species levels at 16 weeks (**Supplementary Figure 7**).

The administration of the Bronze diet induced a significant decrease of *Odoribacter* and an increase of *Prevotellaceae_UCG-001* and *Akkermansia* in both C57BL/6 and Winnie mice, while the *Bacteroides* tends to decrease in Winnie mice fed with the Bronze diet (**Figure 7** and **Supplementary Figure 6**).

Finally, at the species level, the Bronze diet induced in Winnie mice a significant increase of *Prevotellaceae_UCG-001;s_spp.*, as observed at the weaning time; the *Muribaculum;s_spp.* was also increased, while a decrease of *Bacteroides_sartorii*, *Rikenellaceae* spp. and *Odoribacter* spp. was recorded (**Supplementary Figure 7**). In C57BL/6 mice fed with the Bronze diet we observed a significant decrease of *Desulvovibrionaceae* spp., *Oscillospiraceae;g_uncultured;s_spp.* and *Anaerotruncus;s_uncultured_bacterium*, and an increase of *Odoribacter;s_uncultured_bacterium* compared to C57BL/6 mice fed with the Control tomato group (**Supplementary Figure 7**).

## Discussion

IBD genetic transmission is a widely accepted concept, together with the idea that IBD-affected mothers have higher risks of complications during pregnancy and a higher rate of premature delivery (Hashash and Kane, 2015). IBD incidence peaks during reproductive years and 25% of patients experience pregnancy after the IBD diagnosis (Loftus, 2004). The gut microbiota has gained attention during the last decade due to the pivotal role in host intestinal homeostasis, indeed, numerous pathological conditions are associated with an “un-healthy” microbiota composition called dysbiosis. The intestinal colonization is believed to start in utero and adapt to numerous host and environmental factors. The host genetics plays a non-redundant role in shaping the intestinal microbiota (Liso et al., 2019), nonetheless, several different factors further influence the composition of the gut microbiota including cesarean or natural birth, feeding types, weaning, birth environment, and mothers’ microbiota (Nagpal et al., 2017; Raspini et al., 2020). As IBD patients, including mothers during pregnancy, are characterized by reduced α-diversity, it is not surprising that such reduction is transmitted to the offspring (Torres et al., 2020).

Using a murine model of ulcerative colitis called Winnie, we previously compared the microbiome compositions of homozygous mutant mice (Winnie^-/-^) with their C57BL/6 littermates, after weaning from heterozygote Winnie^+/-^ breeders. Heterozygote breeders transmitted dysbiotic microbiota to APC^+/Min^ offspring, resulting in higher rate of CRC development if compared to C57BL/6 breeders (De Santis et al., 2021). We demonstrated that the Winnie offspring displayed significant dysbiosis as early as 4 weeks of age compared with their C57BL/6 littermates (Liso et al., 2019). Furthermore, we investigated the effects on the microbial composition of the Standard, Control tomato and Bronze diets in C57BL/6 and Winnie mice (Liso et al., 2018; Scarano et al., 2018), observing no significant differences between Standard and Control tomato diet among phyla and genera microbial groups. Significant differences were rather observed following administration of Bronze diet, therefore we compared Bronze with Control, instead of Standard rodent diet. Here we aimed to evaluate the possibility to contrast Winnie offspring dysbiosis by feeding the heterozygote Winnie^+/-^ breeders with chows enriched in 1% dried tomatoes. In the present experimental design, we used the Bronze tomato line, enriched in three distinct classes of polyphenols (flavonols, anthocyanins and stilbenoids), and a Control tomato variety (cv. Moneymaker, (Scarano et al., 2018).

Although dietary intervention has been demonstrated to influence gut related microbial functions, without altering both alpha and beta diversity (Li et al., 2016; De Angelis et al., 2020), a study conducted on female rats fed with obesogenic diet demonstrated that diet can alter the gut microbial alpha diversity, as we observed in our study (Bhagavata Srinivasan et al., 2018). Colonic microflora can be influenced by nutritional polyphenols, either directly due to their ability to influence the intestinal ecology (Kumar Singh et al., 2019; Ansary and Cianciosi, 2020; Ullah et al., 2020) and indirectly due to their effect on the host innate and adaptive immune response or iron sequestrating abilities (Cavalcanti et al., 2014; Delvecchio et al., 2015; Chieppa et al., 2017). Our results indicate that nutritional intervention during pregnancy and lactation may influence neonatal microbiota better than dietary intervention in adults. Future human studies will be required to confirm these preclinical observations.

Using Bronze tomatoes as a dietary supplement for the breeding pairs, we immediately noticed a reduction in the mortality rate and an increase in the number of survived Winnie^-/-^. These data, although extremely interesting, require a specific experimental design to increase the number of breeding pairs and prolong the observation period. In light of these preliminary data, we may speculate that the known beneficial effects of a polyphenol enrich diet contributes to reduce mothers’ intestinal and, consequently, systemic inflammation, among the major causes of complication for IBD patients’ pregnancy. These results will be important for the design of future human studies.

Offspring microbiota delivered by mothers under Control tomato and Bronze-enriched diet were analyzed before weaning to evaluate if mothers enriched nutrition during breastfeeding could protect genetically predisposed mice from dysbiosis, mainly due to the increase in the relative abundance of Firmicutes and an increase of Proteobacteria. Of notice, the Bronze enriched diet generally affected mothers’ microbiota favoring a balanced ratio between Bacteroidota and Firmicutes. During lactation, the mothers’ diet has a minor effect on C57BL/6 offspring, while Winnie microbiota is pushed into a “healthier” status. We can’t discriminate if Bronze-enriched mother’s milk has a direct effect on the intestinal microbiota, or if the increase in bioactive compounds reduces inflammation and consequently the intestinal dysbiosis. Most likely, both effects are true as a polyphenol enriched diet has been already demonstrated to improve breastmilk quality and polyphenol content (Khymenets et al., 2016; Tsopmo, 2018). Metabolomics of mother’s milk may help to address this important question. In Winnie offspring, the Bronze diet induced a significant increase of bacterial species belonging to the Lachnospiraceae group and *Prevotellaceae_UCG-001*. The Lachnospiraceae are anaerobic, fermentative bacteria, able to use diet-derived polysaccharides and plant aromatic compounds to produce SCFAs (short-chain fatty acids), like acetate, propionate and butyrate, used from colonocytes as an energetic source, with essential anti-inflammatory properties demonstrated *in vitro* and *in vivo* (Li et al., 2017; Okumura and Takeda, 2018; Vacca et al., 2020). Wang et al. demonstrated an increase in *Lachnospiraceae_NK4A136_group* in a mice model of acute colitis, after administration of probiotics and prebiotics mix (Wang et al., 2019).

An increase of *Prevotellaceae_UCG-001* was associated with a decrease of inflammation and a gut barrier improvement in mice with DSS-induced colitis, treated with a plant-derived decoction (Shen et al., 2020; Zou et al., 2020).

After weaning, mice continued with the same diet of mothers for 12 weeks. The morphological score of the Bronze-enriched diet group had minor improvements if compared to the Control-tomato diet. Similarly, the molecular pathway was similar between mice with the same genetics but different nutritional regimes. However, *Slpi* and *Hmox1* gene expression were increased by the Bronze diet, although not significantly, whilst *Slc40a1* expression was reduced, confirming previous observations on the effects of polyphenols like quercetin on the expression of these markers (De Santis et al., 2016; Galleggiante et al., 2017). At the cellular level, we recorded no significant differences when comparing the cytokines produced from CD4^+^ and CD4^-^ T cells in the MLNs. Nevertheless, a significant reduction in the Foxp3^+^ cells was observed in Winnie mice fed with Bronze diet. Even if Foxp3^+^ cells are involved in inflammatory suppression, their increased number is a sign of ongoing inflammation as demonstrated by several studies. In particular, this transcriptional factor was observed in the intestinal lamina propria of IBD patients compared to healthy control (Wohlfert and Belkaid, 2008; Iacomino et al., 2020). Foxp3 expression was also increased in the colon mucosa during acute and chronic DSS-induced colitis (Yang and Xu, 2016). In Winnie mice, CD4^+^ cells are recruited in the peripheral tissue due to the chronic inflammatory response. This effect is partially reverted in Winnie mice fed with Bronze diet. CD4^+^ cells percentages in the Winnie Bronze MLNs are similar to what observed in the C57BL/6 MLNs, suggesting that intestinal inflammation may be alleviated by the Bronze diet. Furthermore, the microbiota analysis shows a strong correlation between genotype and bacterial content, confirming what we previously observed (Santino et al., 2017; Liso et al., 2018). This genotype-related signature was clearly identifiable at phylum and genus level. These data indicate that long term exposure to polyphenol enriched nutritional regimes may display a reduction in the beneficial effects on microbiota communities selection. On the contrary, this study confirms that short interventions have positive effects on intestinal microbiota of mothers fed with Control or Bronze-enriched tomato diet during pregnancy and lactation and on puppies at 4 weeks.

In conclusion, although our results indicate that prolonged dietary intervention with polyphenol enriched nutritional regimes does not significantly impact the offspring microbiota, we observed significant dysbiosis reduction mainly at weaning time, indicating that the reduction of mothers’ dysbiosis may beneficially affect babies’ microbiota and help preventing/reducing IBD development. Nutrition should become a common adjuvant therapy for dysbiosis mediated inflammatory disorders that may evolve in intestinal or extraintestinal disorders (Ianiro et al., 2020; De Santis et al., 2021; Xu et al., 2021). Based on these results, the present study can contribute to pave the way for future studies based on nutritional interventions designed for IBD mothers.

## Data Availability Statement

Illumina Nextseq550 generated raw sequence reads were deposited at the Sequence Read Archive of the National Center for Biotechnology Information (NCBI). Bioproject accession number: PRJNA718731. Fifthy biosample record IDs: SAMN18559514- SAMN18559563.

## Ethics Statement

The animal study was reviewed and approved by Italian Animal Ethics Committee of Ministry of Health - General Directorate of Animal Health and Veterinary Drugs (DGSAF- Prot. 768/2015-PR 27/07/2015).

## Author Contributions

SDS, ASc and ML conceived and designed the project. SDS, ASc, ML, GV and EC carried out the experiments. FMC performed bioinformatics analysis. MDA and FMC performed statistical analysis. ASi designed nutritional intervention. AL provided funding acquisition. MC and ASa wrote the paper. All authors contributed to the article and approved the submitted version.

## Funding

This work was supported by grants from the Italian Ministry of Health (GR-2011-02347991) Ricerca Corrente 2019 IRCCS “S. de Bellis”, from Apulia Region grant SiCURA “Soluzioni Innovative per la gestione del paziente e il follow up terapeutico della Colite UlceRosA” (KC3U5Y1) and from CNR-DiSBA project NutrAge (project nr. 7022). SDS is funded by PON -Ricerca e Innovazione 2014-2020-: Progetto AIM1801289 - attività 3 - linea 1. ML is founded by M.I.Cro, Associazione Malattie Infiammatorie Croniche Intestinali.

## Conflict of Interest

The authors declare that the research was conducted in the absence of any commercial or financial relationships that could be construed as a potential conflict of interest.
