# Supplementary material for: Polyphenol Enriched Diet Administration During Pregnancy and Lactation Prevents Dysbiosis in Ulcerative Colitis Predisposed Littermates
Source: Front Cell Infect Microbiol. 2021 Jun 9;11:622327. doi: 10.3389/fcimb.2021.622327 (PMC8221423; doi:10.3389/fcimb.2021.622327)
Supplement: Supplementary file 1 [file DataSheet_1.docx]

Supplementary Figures and Tables


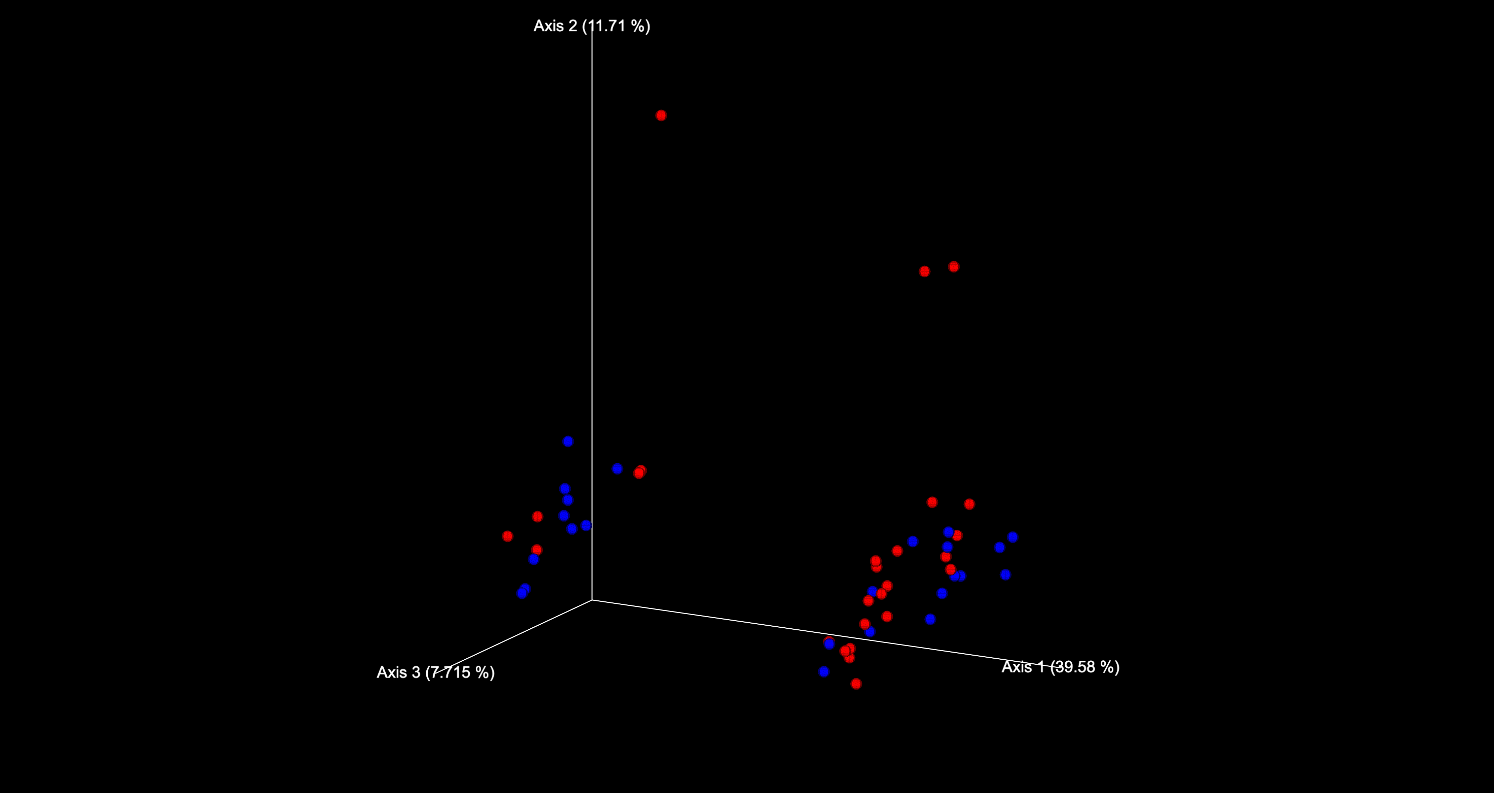


**Supplementary Figure 1.** Phylogenetic beta diversity metric. Unweighted UniFrac estimate of distance between Bronze and Control tomato diets fed mice. Red and blue flagged spots are relative to Bronze and Control samples, respectively. The inferred phylogeny has been computed using q2-emperor QIIME 2 plugin.


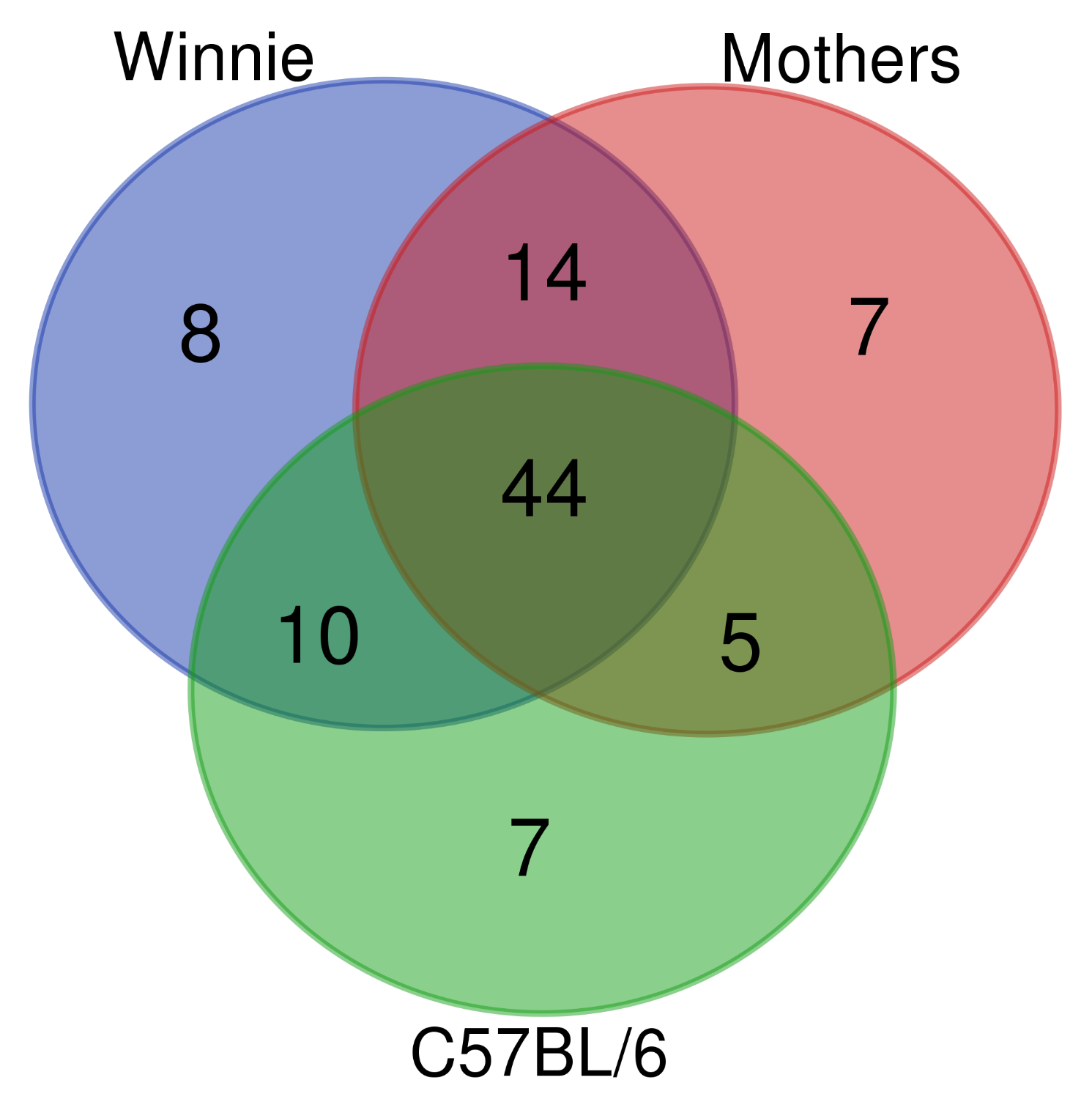


**Supplementary Figure 2.** Mothers and related offspring at 4 weeks (C57BL/6 and Winnie) fed with Control tomato diet Venn plot. Qiime2 computed genus relative abundance values have been transformed in a presence/absence matrix than imported in R. Overlapping feature sets belonging to 4-week Control tomato mice were computed and plotted by using ‘VennDiagram’ package.


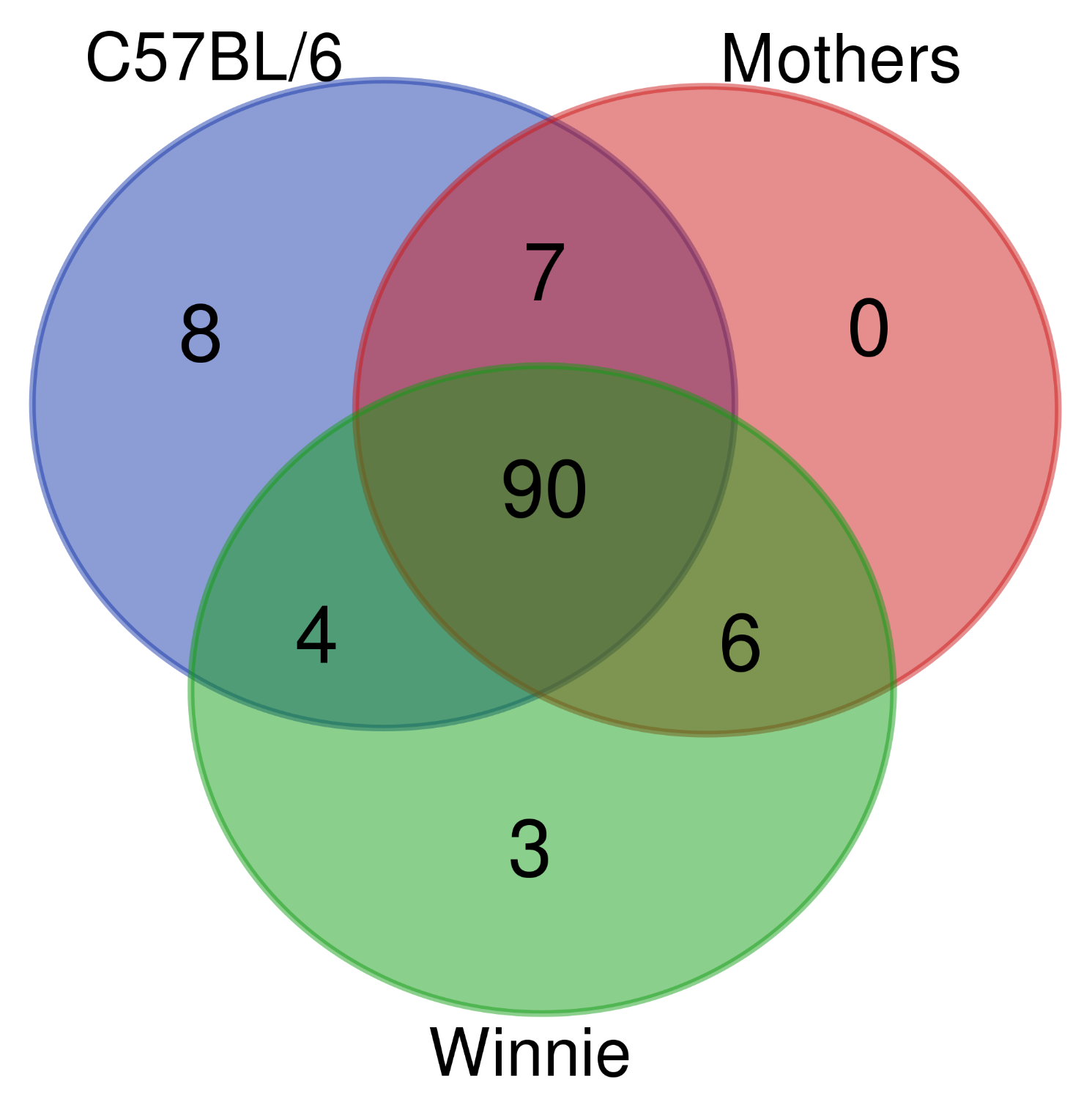
**Supplementary Figure 3.** Mothers and related offspring at 4 weeks (C57BL/6 and Winnie) fed with Bronze tomato diet Venn plot . Qiime2 computed genus relative abundance values have been transformed in a presence/absence matrix than imported in R. Overlapping feature sets belonging to mice that were on a Bronze diet were computed and plotted by using ‘VennDiagram’ package.


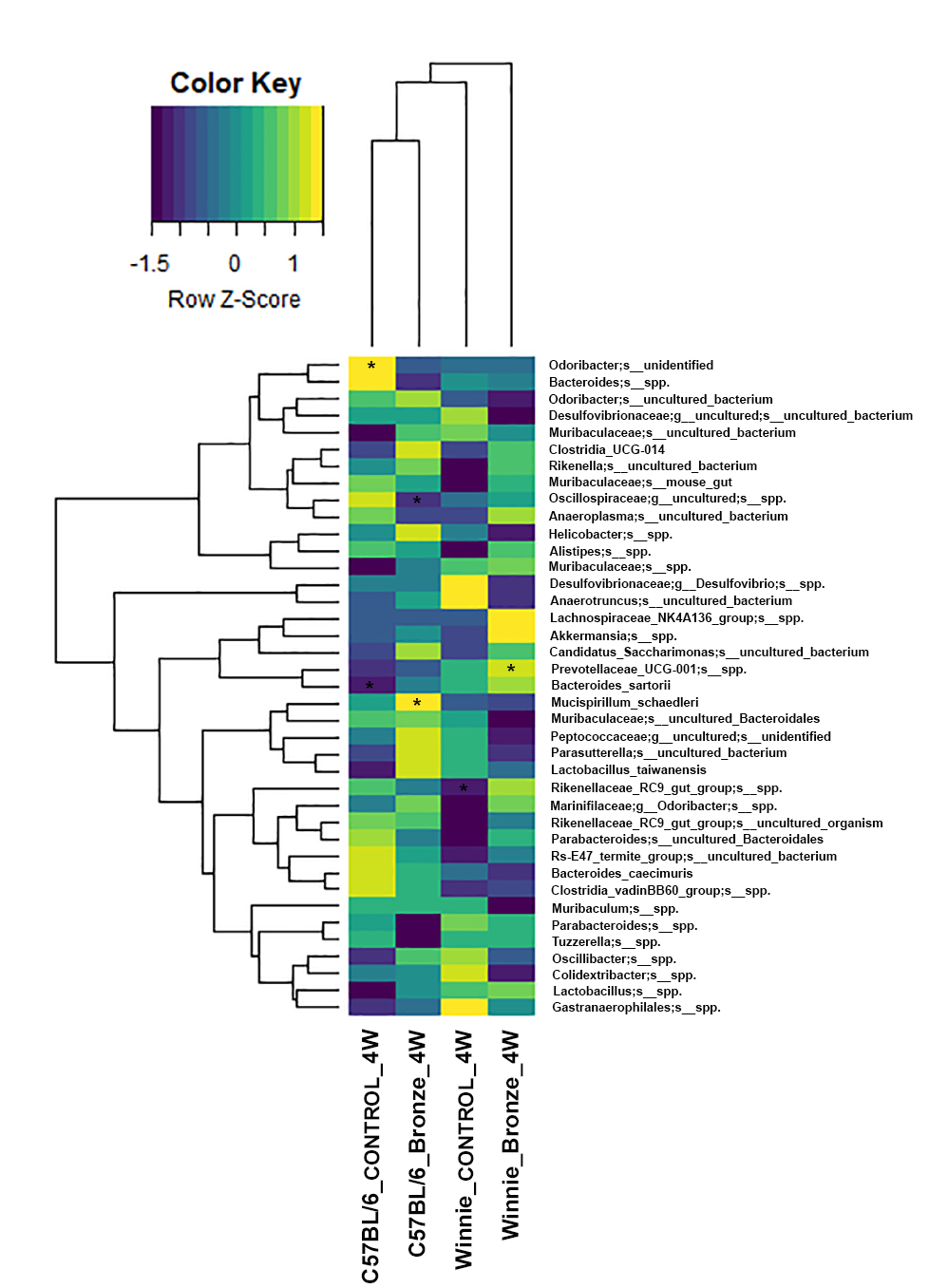


**Supplementary Figure 4**. Heatmap showing the relative abundances of the main species found at weaning time (4 weeks) in the faecal microbiota of C57BL/6 and Winnie mice fed with Control tomato and Bronze diets. Hierarchical clustering was performed using Euclidean distance with average linkage. The relative abundances values (log2 transformed) are represented in scaled expression, denoted as Row Z-Score, and plotted in blue-yellow scale, with blue indicating low values and yellow indicating high values. *p<0.05





**Supplementary Figure 5**. Gene expression of *Tnf*, *Ifnγ*, *Il6*, *Il10*, *Il12b*, *Il17a*, *Hmox1*, *Slpi* and *Slc40a1* in medial colon of C57BL/6 and Winnie mice, fed with Control Tomato- (black bars) and Bronze-enriched diet for 16-weeks (striped bars). * p<0.05; ** p<0.01


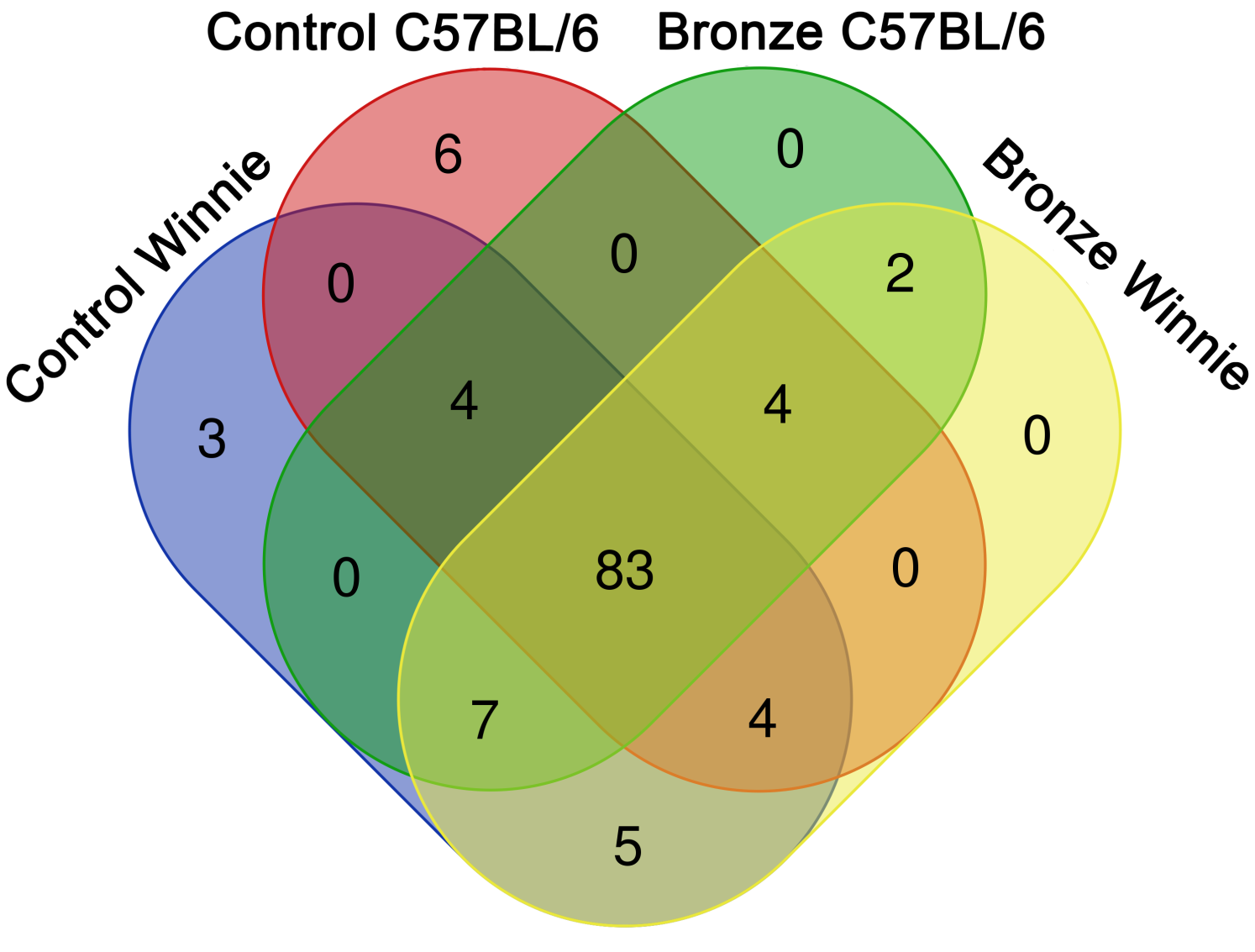


**Supplementary Figure 6.** 16-week Control and Bronze fed mice (C57BL/6 and Winnie) Venn plot. Qiime2 computed genus relative abundance values have been transformed in a presence/absence matrix than imported in R. Overlapping feature sets of 16-week Control tomato and Bronze fed mice were computed and plotted by using ‘VennDiagram’ package.

**
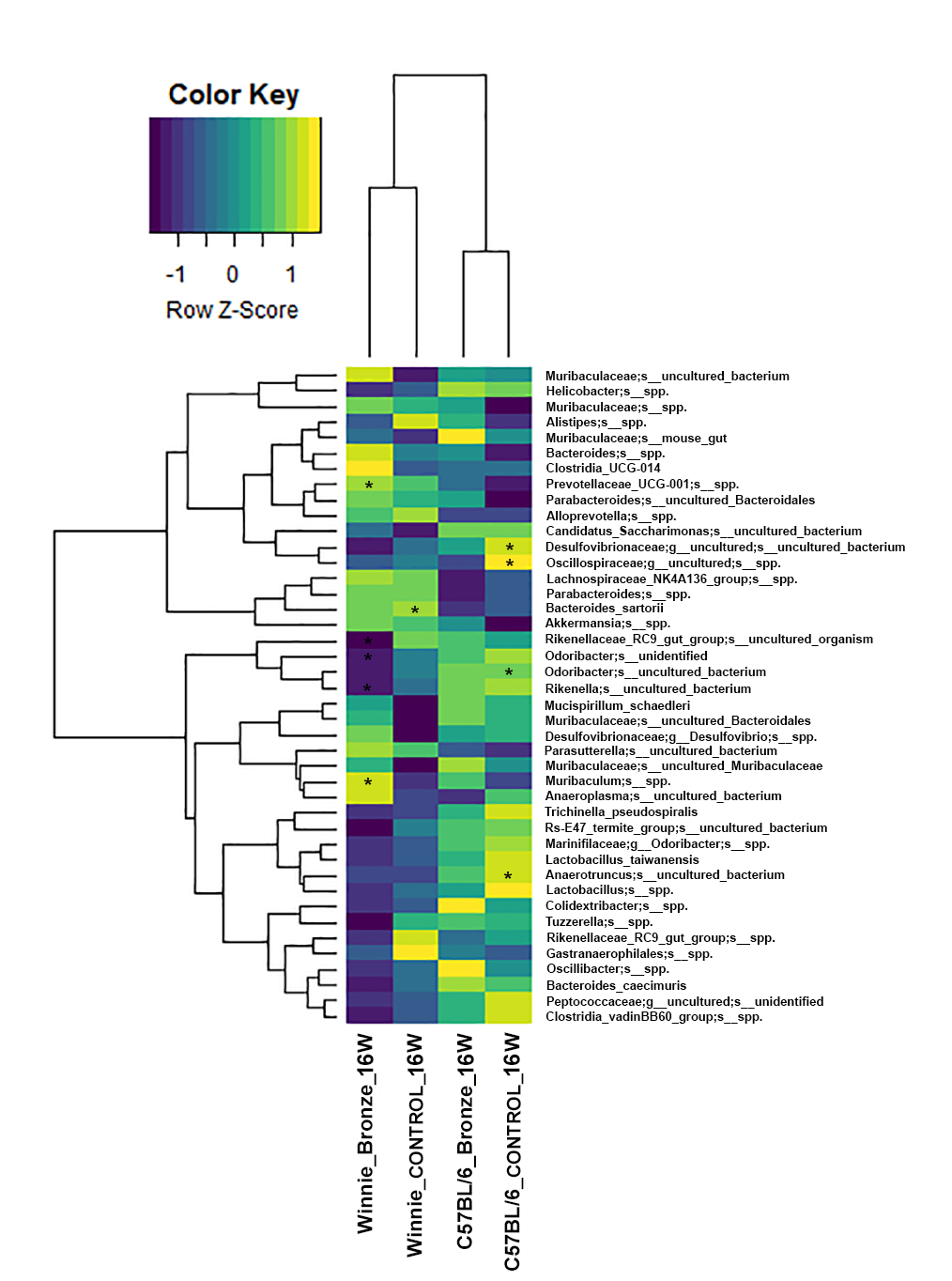
Supplementary Figure 7**. Heatmap showing the relative abundances of the main species found in the microbiota of C57BL/6 and Winnie mice fed with Control tomato and Bronze diets for 16 weeks. Hierarchical clustering was performed using Euclidean distance with average linkage. The relative abundances values (log2 transformed) are represented in scaled expression, denoted as Row Z-Score, and plotted in blue-yellow scale, with blue indicating low values and yellow indicating high values. *p<0.05

**Supplementary Table 1**. Kruskal-Wallis pairwise group comparison for Faith’s PD index both for timing and food variables. H index, p-values and corrected p-values (q-values) are reported for all the pairwise group comparisons.

| **Kruskal-Wallis (pairwise)** |  |  |  |  |
| --- | --- | --- | --- | --- |
| **TIMING** |  | **H** | **p-value** | **q-value** |
| **Group 1** | **Group 2** |  |  |  |
| **16W (n=19)** | **4W (n=21)** | 9.28388 | 0.002312 | 0.023118 |
|  | **III_wean (n=2)** | 5.181818 | 0.022824 | 0.076081 |
|  | **II_wean (n=4)** | 2.631579 | 0.104757 | 0.174596 |
|  | **I_wean (n=4)** | 5.921053 | 0.014961 | 0.074805 |
| **4W (n=21)** | **III_wean (n=2)** | 3.857143 | 0.049535 | 0.123837 |
|  | **II_wean (n=4)** | 0.549451 | 0.458542 | 0.458542 |
|  | **I_wean (n=4)** | 1.406593 | 0.235623 | 0.261803 |
| **III_wean (n=2)** | **II_wean (n=4)** | 3.428571 | 0.064078 | 0.128155 |
|  | **I_wean (n=4)** | 1.928571 | 0.164915 | 0.206144 |
| **II_wean (n=4)** | **I_wean (n=4)** | 2.083333 | 0.148915 | 0.206144 |
| **Kruskal-Wallis (all groups)** |  |  |  |  |
|  | **Result** |  |  |  |
| **H** | 17.1813 |  |  |  |
| **p-value** | 0.00178 |  |  |  |

| **Kruskal-Wallis (pairwise)** |  |  |  |  |
| --- | --- | --- | --- | --- |
| **FOOD** |  | **H** | **p-value** | **q-value** |
| **Group 1** | **Group 2** |  |  |  |
| **BRONZE (n=26)** | **Control (n=24)** | 7.927979 | 0.004868 | 0.004868 |
| **Kruskal-Wallis (all groups)** |  |  |  |  |
|  | **Result** |  |  |  |
| **H** | 7.92798 |  |  |  |
| **p-value** | 0.00487 |  |  |  |
